# *Cryptosporidium hominis* Phylogenomic Analysis Reveals Separate Lineages With Continental Segregation

**DOI:** 10.3389/fgene.2021.740940

**Published:** 2021-10-14

**Authors:** Felipe Cabarcas, Ana Luz Galvan-Diaz, Laura M. Arias-Agudelo, Gisela María García-Montoya, Juan M. Daza, Juan F. Alzate

**Affiliations:** ^1^ Centro Nacional de Secuenciación Genómica CNSG, Sede de Investigación Universitaria-SIU, Medellín, Colombia; ^2^ Grupo SISTEMIC, Departamento de Ingeniería Electrónica, Facultad de Ingeniería, Universidad de Antioquia, Medellín, Colombia; ^3^ Environmental Microbiology Group, School of Microbiology, Universidad de Antioquia, Medellín, Colombia; ^4^ Departamento de Microbiología y Parasitología, Facultad de Medicina, Universidad de Antioquia, Medellín, Colombia; ^5^ Grupo Pediaciencias, Facultad de Medicina, Universidad de Antioquia, Medellín, Colombia; ^6^ Grupo Herpetológico de Antioquia, Institute of Biology, Universidad de Antioquia, Medellín, Colombia

**Keywords:** *Cryptosporidium hominis*, comparative genomics, *de novo* genome assembly, evolution, lineage

## Abstract

*Cryptosporidium* is a leading cause of waterborne outbreaks globally, and *Cryptosporidium hominis* and *C. parvum* are the principal cause of human cryptosporidiosis on the planet. Thanks to the advances in Next-Generation Sequencing (NGS) sequencing and bioinformatic software development, more than 100 genomes have been generated in the last decade using a metagenomic-like strategy. This procedure involves the parasite oocyst enrichment from stool samples of infected individuals, NGS sequencing, metagenomic assembly, parasite genome computational filtering, and comparative genomic analysis. Following this approach, genomes of infected individuals of all continents have been generated, although with striking different quality results. In this study, we performed a thorough comparison, in terms of assembly quality and purity, of 100+ *de novo* assembled genomes of *C. hominis*. Remarkably, after quality genome filtering, a comprehensive phylogenomic analysis allowed us to discover that *C. hominis* encompasses two lineages with continental segregation. These lineages were named based on the observed continental distribution bias as *C. hominis* Euro-American (EA) and the *C. hominis* Afro-Asian (AA) lineages.

## Introduction

*Cryptosporidium* is a ubiquitous apicomplexan parasite with gastrointestinal habitat and a broad range of vertebrate hosts, including humans, other mammals, birds, fish, and reptiles (Zahedi and Ryan, 2020). *Cryptosporidium hominis* and *C. parvum* are the preponderant cause of human cryptosporidiosis around the world, with the former being more frequently found in developing nations (Gilchrist et al., 2018; Tichkule et al., 2021). Cryptosporidiosis is usually a self-limiting infection in immunocompetent individuals. However, vulnerable populations, like immunocompromised individuals (especially those with T cell impairment) and children (particularly those below 5 years old), develop persistent to chronic syndromes, with diarrhea as the principal symptom (Innes et al., 2020). According to the Global Enteric Multicenter Study (GEMS), *Cryptosporidium* was the second cause of moderate-to-severe diarrhea in children from sub-Saharan Africa and South Asia countries; and this infection increased the risk of death in children aged 12–23 months (Kotloff et al., 2013).

*Cryptosporidium* oocysts are excreted in feces by symptomatic hosts. In this stage, the parasite is highly resistant to common disinfection methods like chlorination (Cacciò and Chalmers, 2016). These characteristics might explain why *Cryptosporidium* is a leading cause of waterborne outbreaks globally (Efstratiou et al., 2017). Furthermore, depending on the species, the parasite can be transmitted via direct person-to-person contact, indirect (food, water, or fomites), or zoonotic routes (Cacciò and Chalmers, 2016).

Currently, 42 species of *Cryptosporidium* are recognized (Zahedi and Ryan, 2020), with *C. parvum* and *C. hominis* being responsible for greater than 90% of human infections (Feng et al., 2018). Geographic differences in species distribution have been reported, with *C. hominis* as the leading species in human cases in developing countries (Xiao and Feng, 2008; Ryan et al., 2014; Xiao and Feng, 2017). While *C. hominis* is associated with a predominant anthroponotic transmission*, C. parvum* presents a zoonotic transmission route with livestock as the primary source of infection (Nader et al., 2019). In developed European nations, *C. parvum* infections are more common in rural areas with low human population density and activities related to livestock and agriculture (Lake et al., 2007; Pollock et al., 2010). By contrast, *C. hominis* has a narrow host range showing a specialization trend toward human hosts. Although it can successfully infect other mammals, it produces mild and asymptomatic infections (Widmer et al., 2020). Subtyping characterization through gp 60 gene analysis reveals more than ten subtype families, with six of them as the most common in humans (Ia, Ib, Id, Ie, If, and Ig), being the IbA10G2 subtype the predominant and most virulent, widely distributed in both developing and developed countries, and frequently associated with outbreaks worldwide (Feng et al., 2018).

*Cryptosporidium hominis* is an unculturable parasite. This condition implies that the only possibility to obtain genomic DNA of the parasite is to extract it from stool samples of infected hosts. *Cryptosporidium* oocysts in human feces are usually present at low numbers, requiring specific procedures to capture them. Despite the purification step, DNA of the intestinal microbiota is often abundant, requiring a metagenomic bioinformatics strategy to study the *Cryptosporidium* genome. A metagenome assembly is performed to start the genome reconstruction. Then, *C. hominis* genome scaffolds should be selected using informatics tools. Due to the risk of *Cryptosporidium* coinfections, different species, or isolates, additional quality control steps are needed to avoid contaminated/chimeric genome reconstructions (Isaza et al., 2015).

In this study, we conducted a comparative analysis of the publicly available genomic data of *C. hominis* under normalized conditions with the aim to have a better understanding of the quality of the generated data in the last decade. Since all these genomes come from a methodology more like a metagenomic approach, additional analyses were performed to detect *Cryptosporidium* genome mixtures present in one individual.

We also used phylogenetic tools to reconstruct the evolutionary history of ninety-nine *C. hominis* genomes collected from human individuals in five continents: America, Europe, Asia, Africa, and Oceania. Our phylogenomic analysis showed that *C. hominis* species encompasses two lineages with phylogeographic structure, with one clade composed mainly of European and American isolates, while the other mainly with African and Asian isolates. The two Oceania representatives were grouped into the Euro-American lineage.

## Materials and Methods

### Genome Data From Sequence Read Archive-SRA Public Database

One hundred nineteen *C. hominis* Next-Generation Sequencing (NGS) genome projects with shotgun reads were selected and downloaded (Supplementary Table S1) from the Sequence Read Archive-SRA database. Genome projects based only in 454 Technology were excluded. The raw read data were directly download from the SRA database using the fastq-dump tool with the split option activated.

As outgroups, another 15 genomes were used: *C. cuniculus* (UKCU5: PRJNA492839, UKCU2: PRJNA315496), *C. parvum* (UKP2:PRJNA253836, UKP3:PRJNA253840, UKP4:PRJNA253843, UKP5:PRJNA253845, UKP6:PRJNA253846, UKP7:PRJNA253847, UKP8:PRJNA253848, UKP14:PRJNA315506, UKP15:PRJNA315507), and *C. meleagridis* (UKMEL1:PRJNA222838, UKMEL3:PRJNA315502, UKMEL4:PRJNA315503).

### Generation of the New Colombian *Cryptosporidium hominis* Genome Reference UDEAa567

*C. hominis* oocysts were purified from a fecal sample collected from an HIV Colombian female patient by flotation in a saturated sodium chloride solution (Kar et al., 2011). Parasite diagnosis was previously confirmed by Kinyoun stain. Species and subtype identification was done by a nested PCR and sequence analysis of the small-subunit (SSU) rDNA gene and 60 kDa glycoprotein gene (IbA10G2), respectively. Purified oocysts were resuspended in PBS and quantified using a Neubauer chamber slide and light microscopy. The sample was stored at 4°C until DNA was extracted. DNA extraction was performed using the kit NORGEN Stool DNA Isolation (CAT 27600), following the manufacturer’s instructions, with previous freeze-thaw cycles (each for 2 min) in liquid nitrogen and a water bath at 37°C. A solution with approximately 1.85 × 10^8^ oocyst was used for DNA purification. DNA obtained was quantified by light absorption at 260 nm (NanoDropTM, Thermo Scientific) and with PicoGreen^®^ reagent. The genome was sequenced using Illumina NOVASEQ 6000 instrument at Macrogen (Seoul, Korea). Paired-end reads of 150 bases were generated and deposited at the SRA database under the accession number SRR14522748.

### *Cryptosporidium hominis De Novo* Genome Assembly

Genomic reads from each experiment were independently assembled using SPADES v3.14.1. First, reads ends were cleaned with rapifilt (homebrew program) filtering at Q30 and only keeping reads with a minimum of 50 bases. Then, assembly was performed using SPADES parameters: -careful -t 40 -m 160 -k 33,55,77,99.

Each assembly was filtered to excluded contaminating sequences using BLASTN (v2.10.1+). Only those longer than 1,000 bases and with a bit score greater than 300 were kept in each *C. hominis* assembled genome dataset. The bit score was obtained mapping the scaffolds to the *C. parvum* IOWAII genome reference (CryptoDB v 52), using BLASTN with parameters -evalue 1e-30 -num_alignments 5.

The genomes stats (Total length of sequence, Largest contig, N50 stats, Total number of sequences) were obtained using an in-house python script (see Supplementary Material). The “AltSNPs” (alternate single nucleotide polymorphisms) and “Coverage” of the assembly, were obtained by mapping the cleaned reads of each experiment against the assembled genome, using bowtie2 (version 2.4.1) with default parameters and using samtools (v1.10) view -F 3584 to keep only mapped reads. The coverage was obtained using samtools coverage and obtaining the mean and median of the mean depth of each scaffold. While the SNVs were obtained by counting the number of variants from the Variant Call Format (VCF) created as bcftools mpileup--redo-BAQ--in-BQ 30--per-sample-mF--skip-indels, then bcftools call--multiallelic-caller--variants-only-Ov and then bcftools view -i “%QUAL ≥ 30.”

The SNVs and Identity vs. *C. hominis* UDEA01 were obtained comparing each genome with the *C. hominis* UDEA01 using DNAdiff (version 1.3) and getting the TotalSNPs and AvgIdentity from the.report file. The *C. hominis* genomes that had less than 8,167,000 assembled bases and with more than 3,825 AltSNPs were dropped as they did not meet basic quality parameters, to obtain the selected genomes that are further analyzed. We run BUSCO v5.2.2 (Simão et al., 2015) with parameters “busco-m geno-l coccidia_odb10 -i” for each selected genomes, including outgroups.

The indels vs *C. parvum* IOWAII were obtained using nucdiff (v2.0.3), with default parameters for each selected genome and the additional *C. parvum*, *C. meleagridis*, and *C. cuniculus* genomes. Using an in-house python script the indels were extracted from “_query_snps.gff” files. The principal component analysis (PCA) analysis was created with python’s sklearn package and plotted with matplotlib.

### Phylogenomic Analysis

The selected *C. hominis* genomes, together with the *C. parvum, C. meleagridis* and *C. cuniculus* genomes were used to infer a phylogenetic tree. The sixty one neutrally evolving genes of *C. parvum* described by Nader et al. (2019) were extracted from each genome. Each gene from all genomes were aligned using MAFFT (version v7.475) with parameters--inputorder--adjustdirection--anysymbol--auto. All the aligned genes were concatenated using catsequences. These aligned sequences were used to infer a maximum likelihood tree using IQTREE2 (2.1.2 COVID-edition for Linux 64-bit) with parameters -B 5000-T AUTO -m MFP+MERGE -rcluster 10 (Lanfear et al., 2012; Lanfear et al., 2014).

Best-fit models selected according to BIC for each coding gene were: K3Pu + F + G4:cgd1_1450 + cgd4_4440 + cgd6_2560 + cgd7_1810 + cgd7_2600 + cgd8_3560,TPM3u + F +G4:cgd1_1730 + cgd1_3650 + cgd1_640 + cgd6_2720 + cgd6_5300 + cgd7_1270 + cgd7_340 + cgd7_890,TIM2 + F + G4:cgd1_2000 + cgd1_3790 + cgd2_2470 + cgd3_4230 + cgd4_2820 + cgd5_2250 + cgd5_4240 + cgd7_2340 + cgd8_2850,TN + F + G4:cgd1_3780 + cgd3_1720 + cgd4_3800 + cgd4_4360 + cgd6_4090 + cgd6_4280 + cgd6_5370 + cgd8_2080 + cgd8_830,TN + F + G4:cgd2_180 + cgd2_3630 + cgd8_1960 + cgd8_3030 + cgd8_5310,HKY + F:cgd2_2060 + cgd5_1340,TN + F + G4:cgd2_3110 + cgd3_1010,K3Pu + F + G4:cgd2_3810 + cgd2_940 + cgd3_380 + cgd4_2620 + cgd5_2890 + cgd7_1330 + cgd7_3550,TPM3 + F + G4:cgd3_2600 + cgd5_3600 + cgd6_2100,HKY + F + I:cgd3_3070 + cgd3_3650 + cgd4_2210 + cgd5_2730,TPM3 + F + G4:cgd3_3310 + cgd5_2700,HKY + F + G4:cgd4_2180 + cgd4_370 + cgd5_1860,TPM2 + F + G4:cgd8_140

We built a haplotype network using the minimum spanning network algorithm (Bandelt et al., 1999) and implemented in PopART (Leigh and Bryant, 2015). We included 95 genomes and excluded four genomes (Chom_EU_SWEH8, Chom_EU_SWEH5, Chom_AFRICA_SWEH3, Chom_AFRICA_SWEH4) because their long branches inferred in the phylogeny, resulting most likely from sequencing errors instead of true genetic variation. The dataset included 118 segregating sites across the 61 genes. The complete step by step analysis pipeline can be found in Supplementary Material.

### Statistical and Graphical Analysis

Statistical analysis and graphics were done in R [R version 4.0.4 64, x86_64-apple-darwin17.0 (64-bit)] and R studio (Version 1.2.1335, Macintosh; Intel Mac OS X 10_16_0). Boxplots were generated in R with the function “boxplot.” Central tendency measures and inter-quartile ranges (IQR) were calculated with R functions “mean,” “median,” “quantile,” and “IQR.”

## Results

### *Cryptosporidium hominis* Genome Quality Analysis

One hundred and nineteen *Cryptosporidium hominis* genomes were included in the present study. A new Colombian *C. hominis* genome with the code UDEAa567 was also generated following *C. hominis* UdeA01 strategy (Isaza et al., 2015): the oocysts were enriched using a flotation protocol from a stool sample from an HIV + infected individual and then the NGS sequencing was performed using an Illumina Novaseq 6,000 instrument. *C. hominis* UDEAa567 assembly genome size was 9,052,438 bp. with an N50 value of 93,658 bp. The mean and median read depths were 10.2X and 10.5X, respectively. The UDEAa567 has 99.93% genome identity and 2,655 Single nucleotide variants (SNVs) respect *C. hominis* UDEA01.

To normalize the analytical conditions for all *C. hominis* genomes, the processing started with the original raw read data, and then they were all assembled using the same strategy. The *C. parvum* IOWA II genome (version 52) was downloaded from CryptoDB. All the read sequences used (*C. hominis*, *C. parvum*, *C. cuniculus*, *C. meleagridis*) are publicly available at the NCBI/SRA website (see *Materials and Methods*).

**FIGURE 1:**
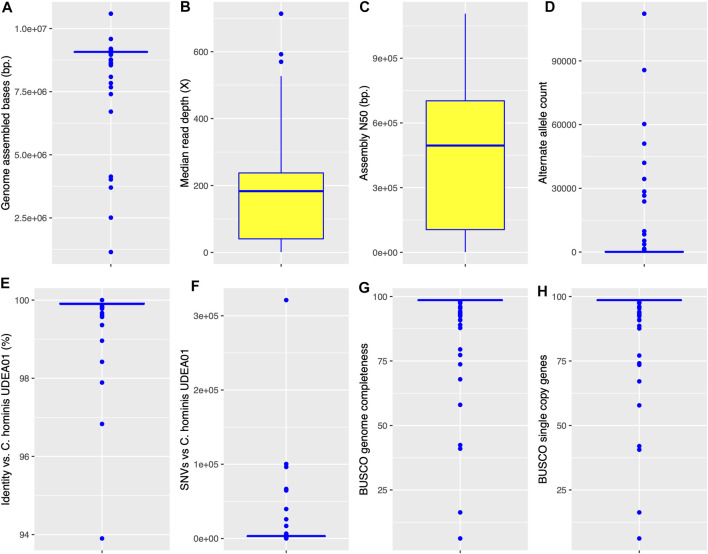
Boxplots representing the descriptive statistics of the *de novo* assembled *C. hominis* genomes. The box represents the interquartile range, IQR 25th -75th percentile. The line represents the 50th percentile. Whiskers denote the 0th and 100th percentile. **(A)** *Cryptosporidium* genome assembled bases. **(B)** Scaffold median read depth. **(C)** *Cryptosporidium* assembly N50 value. **D**. Scaffold positions with an alternate allele. **(E)** Assembly genomic nucleotide identity against *C. hominis* UDEA01 reference. **(F)** Single nucleotide variants (SNVs) detected in each *C. hominis* assembly against *C. hominis* UDEA01 reference. **(G)** BUSCO genome completeness. **(H)** BUSCO single-copy genes.

The *C. hominis* genome projects were executed in the last 10 years with different Illumina instruments, different library preparation kits, and were conducted in different laboratories around the world. A small set of these genomes were generated on the ION TORRENT platform. To assess the quality of the assemblies, their general metrics were compared in Figure 1: total assembled bases, median sequencing depth, assembly N50 values, genome nucleotide identity with *C. hominis* UDEA01, alternate allele count, BUSCO genome completeness, and BUSCO single-copy genes (Supplementary Table S1). The *Cryptosporidium* assembled genome sizes ranged between 1,148,020 and 10,587,153 Mbp. Despite the broad range of the assembled bases metric, a very narrow dispersion range (an IQR of 21,974 bases) was observed around the median, 9,073,830 bp (Figure 1A). By contrast, the observed median sequencing depth and its IQR were both very variable. The depth ranged from 1.1× to 714×, with a median value of 183× and an IQR of 197 (Figure 1B).

The assembly N50 value also showed a very broad range, from 1,302 to 1,106,561 bp. The median N50 value was 495,148 bases with an IQR of 597,468 (Figure 1C). Furthermore, the global nucleotide identity of all genomes against the *C. hominis* UDEA01 reference was calculated. They have a median identity of 99.9% and an IQR of 0.02% (Figure 1E). Only 10 genomes had a global identity value below 99.7% (Chom_EU_4127, Chom_AFRICA_SWEH7, Chom_EU_SWEH9, Chom_EU_6940, Chom_EU_6946, Chom_EU_6925, Chom_EU_6934, Chom_EU_4120, Chom_EU_4128, Chom_EU_4118). These genomes are poor-quality references since they are outliers in either assembled genome size or alternative allele count metrics (Supplementary Table S1).

To assess the possible *Cryptosporidium* coinfections, the reads of each isolate were mapped to its respective assembly and alternate alleles were counted. The observed median number of genomic positions with an alternate allele was 83, with an IQR of 196 (Figure 1D). Eighty-five percent of the *C. hominis* isolates showed alternate allele counts below 470. Then, to estimate the genetic variations among the *C. hominis* genomes, we counted the number of SNVs against the *C. hominis* UDEA01 reference. This analysis showed a median value of 3,324 SNVs with an IQR of 831 (Figure 1F). Ninety percent of the *C. hominis* genomes had less than 4,000 SNVs regardless its continental origin.

We analyzed the *C. hominis* genomes with BUSCO using the coccidia ODB10 database. The BUSCO analysis shows the same median and IQR values for both completeness and single-copy metrics, 98.6 and 0.2%, respectively (Figures 1G,H). The duplicated BUSCO median value was 0 with an IQR of 0. All *C. hominis* genomes, except 1 (Chom_EU_6925), showed duplicated values below 1%. Finally, the BUSCO fragmented metrics showed a median value of 0.4% with an IQR of 0.2% (Supplementary Table S1).

**FIGURE 2:**
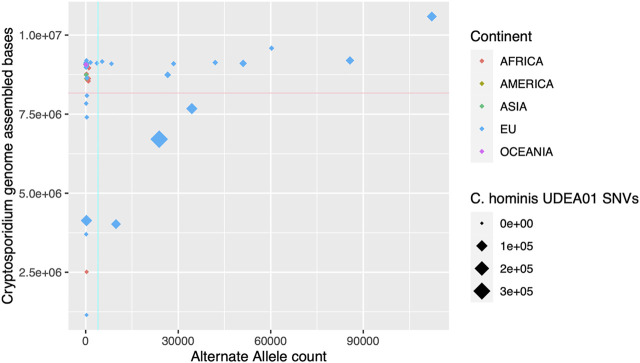
Graphical representation of the *de novo* assembled *C. hominis* genome size and alternate allele detection. In the x-axis, alternate allele count for each genome. In the y-axis, *Cryptosporidium* assembly size in bases pairs. The continent where the parasite was isolated is represented in colors. The size of the rhombus depicts the single nucleotide variants (SNVs) counts against the *C. hominis* UDEA01 genome reference. The cyan and pink lines indicate the thresholds established for low-quality genomes, alternate allele count = 3,992, and assembled genome size = 8,166,447 bases, respectively.

The genomic descriptive measurements showed outliers in several statistic indices, indicating the presence of poor-quality genomes. To filter out them, we set two thresholds: assembled genome size and alternate allele count. The first parameter is directly related to the coverage achieved for each genome and failing to reach a minimum threshold will affect the assembly size and also the accuracy of the assembly. The second parameter, alternate allele count, might indicate the presence of more than one *Cryptosporidium* genome in the human stool sample. The proposed thresholds rationale are: 1) Assembly size > 8,166,447 bp., which corresponds to 90% of the median value of *C. hominis* assembled genome size. We think that a representation of 90% of the expected assembly genome size is enough for subsequent comparative or phylogenetic analysis and is equivalent to a BUSCO genome completeness score of >90% (Supplementary Table S1); and 2) AltSNVs threshold of 3,992 SNVs, which corresponds to the percentile 90th of the observed HQ SNVs of the analyzed *C. hominis* genomes (Figure 2). Applying these two thresholds, 20 genomes were excluded for further analysis, 18 with European origin and 2 with African origin (Chom_EU_SWEH9, Chom_EU_UKH6, Chom_EU_SWEH13, Chom_EU_SWEH11, Chom_EU_H58, Chom_EU_6946, Chom_EU_6945, Chom_EU_6942, Chom_EU_6940, Chom_EU_6939, Chom_EU_6935, Chom_EU_6934, Chom_EU_6925, Chom_EU_6919, Chom_EU_4128, Chom_EU_4127, Chom_EU_4120, Chom_EU_4118, Chom_AFRICA_SWEH7, and Chom_AFRICA_SWEH2). After filtering, the number of genomes per continent was Africa, 33; America, 6; Asia, 29; European Union, 49; Oceania, 2.

### Phylogenomic Analysis of the *Cryptosporidium hominis* Species

Following the previous work published by Nader et al. (2019), 61 genes identified as neutrally evolving in *C. parvum* and *C. hominis* were used for the phylogenomic analysis. After filtering out the low-quality *C. hominis* genomes, ninety-nine genomes were kept for the phylogenetic reconstruction. As outgroups, 15 genomes were used: two *C. cuniculus* (UKCU5 and UKCU2), 8 *C. parvum parvum* (UKP2, UKP3, UKP4, UKP5, UKP6, UKP7, and UKP8), three *C. parvum anthroponosum* (UKP14 and UKP15), and three *C. meleagridis* (UKMEL1, UKMEL3, and UKMEL4).

**FIGURE 3:**
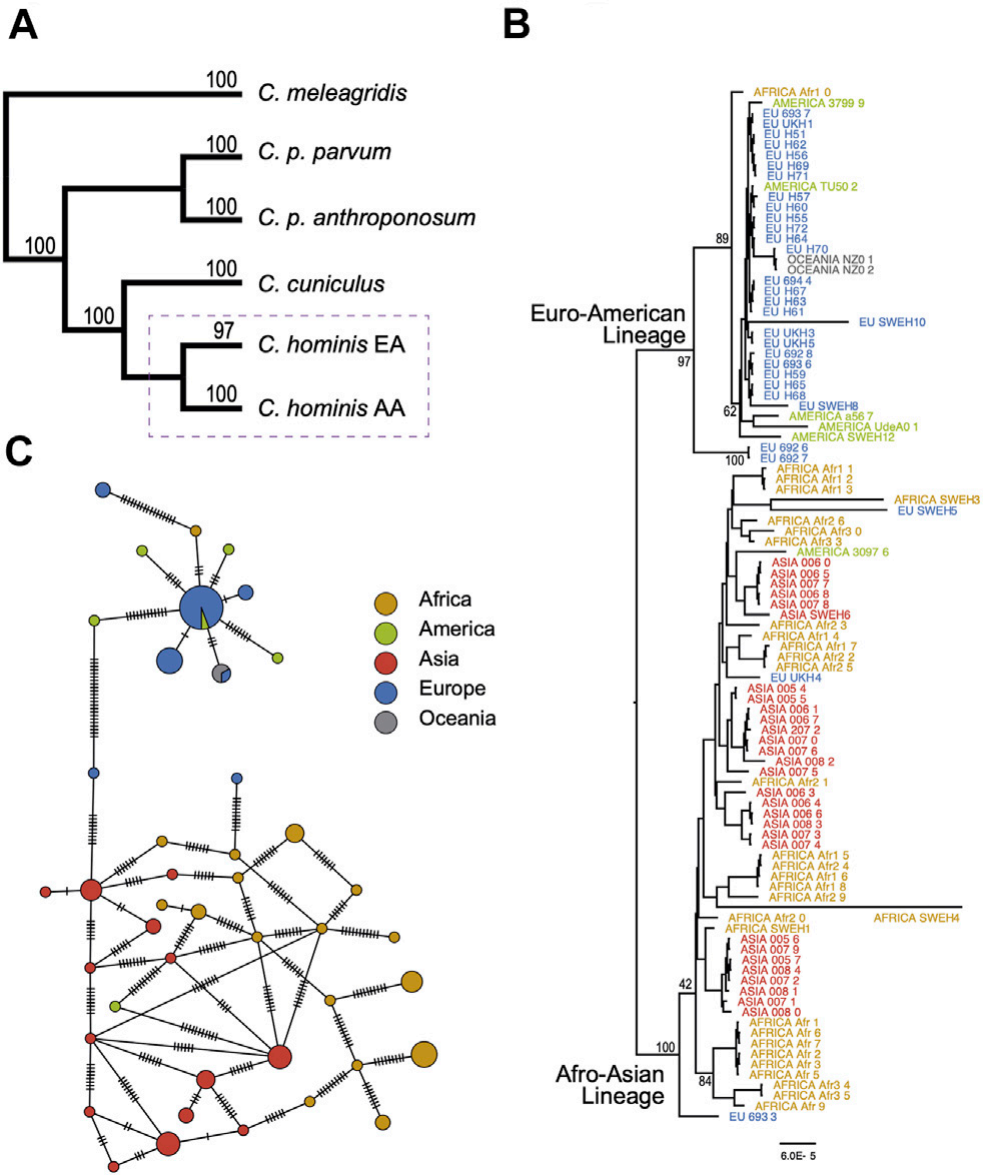
Evolutionary relationships among species and populations of *Cryptosporidium hominis*. **(A)** Summary of main phylogenetic relationships among the main human-infecting *Cryptosporidium* species. The complete tree is provided in the Supplementary Material. The dashed rectangle is expanded in **(B,C)**. **(B)** Subtree depicting the phylogenetic relationships among *C. hominis* isolates from five continents. Tree inference based on 61 coding genes and 140,425 sites. **(C)** Haplotype network based on 116 segregating sites across 61 coding genes (see methods for details). The size of the circles is proportional to the number of individuals with each haplotype. The cross lines represent the number of expected mutations among haplotypes.

A maximum-likelihood tree was constructed encompassing 114 tips. The total matrix of the 61 aligned genes encompasses 140,425 sites. BIC partitions were reduced to 13 (see *Materials and Methods*). The consensus tree has a Log-likelihood of −266,302.24. The basal branches recreate the previously described topology for the analyzed species, with *C. meleagridis* defined as the outgroup. The *C. parvum* subspecies clades *C. parvum* parvum and *C. parvum anthroponosum* are reciprocally monophyletic with 100% bootstrap support. The clade *C. hominis* comprises two well-supported lineages with strong continental bias: One lineage representing isolates from Asia and Africa (100% nodal support), and the other lineage representing genomes isolated from European or American infected humans (97% nodal support). The two isolates from Oceania are part of the European-American clade (Figure 3A). From now on, these two lineages will be referred to as the *C. hominis* Euro-American lineage (EA) and the Afro-Asian lineage (AA). The consensus tree shows very well-supported basal nodes but low support at the terminal branches due to low divergence among closely related genomes (Figure 3B). It is also noteworthy that the *C. hominis* EA lineage is populated by Ib genotypes, mostly IbA10G2/IbA12G2, except for *C. hominis* UdeA01, which has been classified with the genotype Ie.

Four terminal branches showed exceptional longer branch lengths, one in the AA lineage, and three in the EA lineage. It is also particular to see that these four samples come from the same study and were sequenced with the same instrument, the ION TORRENT.

To further confirm the lineage segregation in *C. hominis*, haplotype analysis was performed based on the 61 neutrally evolving genes. Haplotype network is congruent with the two main lineages (Euro-American and Afro-Asian lineages, Figure 3C). However, we did not find structure within these two lineages, and haplotype diversity appears to be higher in the Afro-Asian lineage.

### Genomic Deletions Analysis

Indel events are common in *Cryptosporidium* parasites (Arias-Agudelo et al., 2020). To assess the genomic deletion and insertion profiles within *C. hominis* species, genomic alignment of the assemblies against the *C. parvum* IOWA II reference was performed and analyzed. Insertion and deletion events were treated independently to give more clarity.

**FIGURE 4:**
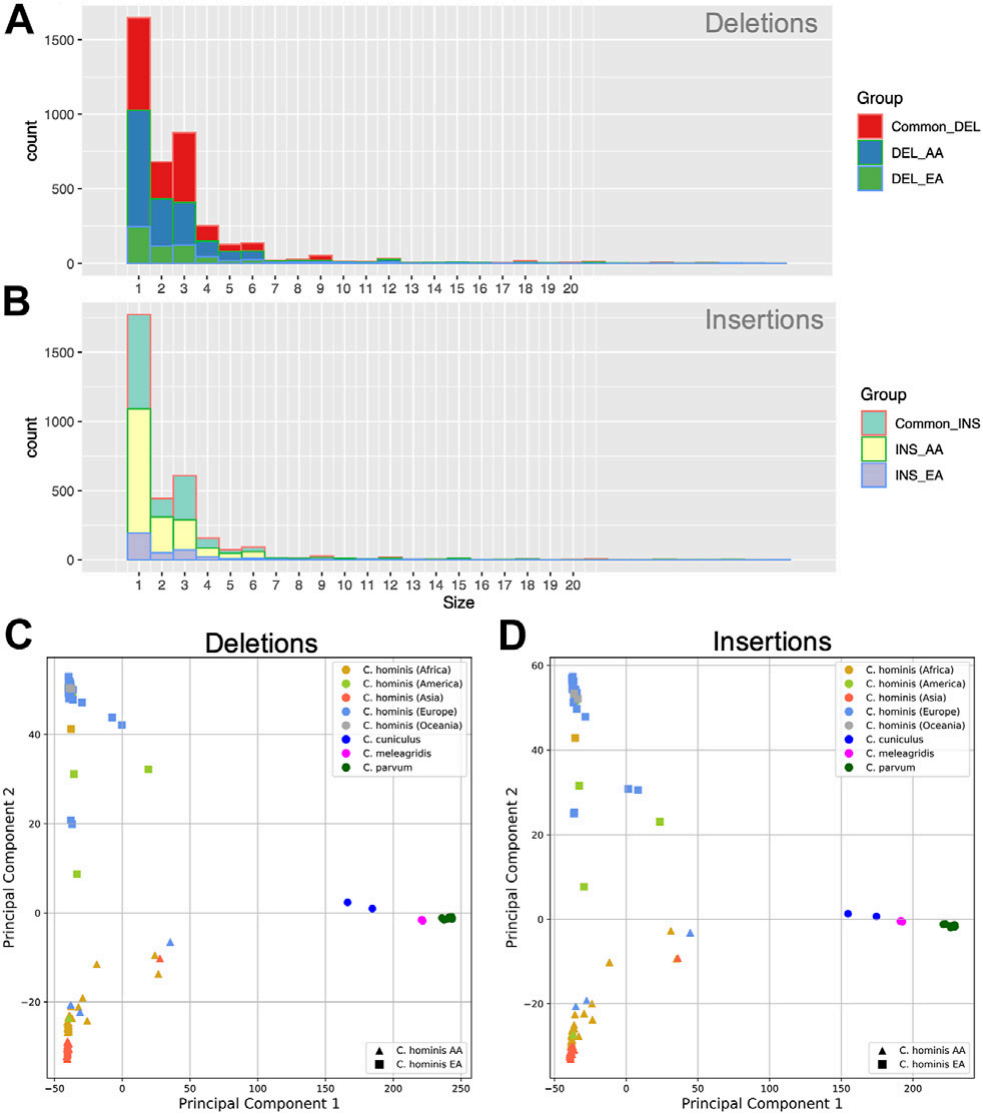
Genomic insertion and deletion events in *C. hominis* lineages. **(A)** Genomic deletion event length (base pair) frequency common to both lineages (Common_DEL), EA and AA. DEL_AA: Specific to the AA lineage. DEL_EA: specific for the EA lineage. **(B)** Genomic Insertion events length (base pair) frequency common to both lineages (Common_INS), EA and AA. INS_AA: Specific to the AA lineage. INS_EA: specific for the EA lineage. **(C)** Principal component analysis (PCA) of the genomic deletion events detected in *C. hominis*. **(D)** Principal component analysis (PCA) of the genomic insertion events detected in *C. hominis*.

Deletion events were more common than insertions and spanned from 1 to 78 bases. The most frequent loss harbored a single base and was followed in frequency by the loss of three bases (Figure 4A). All the *C. hominis* genomes displayed a set of 3,958 shared deletion events. Furthermore, there are 609 specific deletion events in *C. hominis* EA lineage, and 2,338 specific deletion events in AA lineages.

Like the deletions, the insertion events spanned from 1 to 68 bases, being, in order, more frequent those that involve one and three bases (Figure 4B). Most of the insertion events were shared between both lineages (*n* = 3,272). Nonetheless, each lineage displayed specific insertion events. The AA lineage has 1,996 common insertions, while the EA lineage has 380.

Indel events may help to understand the relationship among the different parasite clades. With the aim to test the informative signal of the indel event profile of each genome, a Principal component analysis (PCA) analysis of the studied genomes was performed. As can be seen in Figures 4C,D, deletions or insertion event profiles segregate the parasites according to species and the lineage of *C. hominis*.

## Discussion

We are at the dawn of a new era in microbiology. Nowadays, genomes of pathogens are analyzed directly from infected tissues or other human-derived samples like feces. Next-generation sequencing technologies not only fostered, in general, the high-resolution analysis of life on Earth but also prompted the genomic analysis of non-culturable pathogens like *C. hominis*. In this apicomplexan, we can observe how metagenomic analyses nurtured the advance in comparative and evolutionary genomics. Hundreds of reference genomes were generated in the last decade using the oocysts enrichment and metagenomic shotgun sequencing strategy. (Guo et al., 2015; Hadfield et al., 2015; Isaza et al., 2015; Ifeonu et al., 2016; Sikora et al., 2017; Gilchrist et al., 2018; Morris et al., 2019; Nader et al., 2019; Xu et al., 2019; Arias-Agudelo et al., 2020; Tichkule et al., 2021).

This metagenomic strategy poses challenges like the need for new standardized informatics methods to validate genome quality and purity since the infected individual could harbor a mixture of *Cryptosporidium* parasites: either different lineages of the same species or even multispecies infections. Furthermore, this strategy offers a great opportunity to study pathogens without the bias of artificial selection *in vitro* cultures and observe their genomes in natural settings under the complex conditions where they must co-exist and compete with other microorganisms.

The *C. hominis* genomes, sequenced so far, were generated in very dissimilar conditions, with different oocysts isolation protocols, and sequenced with different instruments in the last decade (Hadfield et al., 2015; Isaza et al., 2015; Ifeonu et al., 2016; Sikora et al., 2017; Gilchrist et al., 2018; Morris et al., 2019; Nader et al., 2019; Tichkule et al., 2021). The results presented in this work show that descriptive genome assembly statistics differ significantly, although some metrics have very narrow dispersion values enabling a rapid way to detect outliers. For instance, *C. hominis* assembled genome size has a narrow dispersion around the median value of 9,074,262 bp (IQR 20688 bp.), regardless of its continental origin or the NGS platform used. Additionally, the global nucleotide identity and SNVs count against the *C. hominis* UDEA01 also have a narrow dispersion close to their respective median values. This might be used as a marker for intraspecies boundaries for *C. hominis* genome around 99.7% identity and 4000 SNVs.

The median sequencing depth and the N50 values show the highest dispersion ranges. Median sequencing depth depends mainly on two main factors: The efficiency of the *Cryptosporidium* oocysts enrichment process, and the sequencing scale (number of reads generated). Even after an efficient enrichment process, *C. hominis* oocysts will be accompanied by intestinal microbiota, mainly bacteria. The more bacteria present in the sample, the fewer reads that will be available to support the target *Cryptosporidium* genome.

Insufficient sequencing depth might generate smaller assembled genome sizes, less accurate scaffolds, lower N50 values, and more fragmented genome models. Another situation that should be considered is that mixed parasite populations in one infected individual might lead, even under sufficient sequencing depth conditions, to more fragmented assemblies. In the case of samples with mixed *Cryptosporidium* species, it might be possible to observe outlier assemblies with larger-than-expected genome sizes and higher counts of alternate alleles.

It is noteworthy to mention that the phylogenomic analysis also enables to detection of outlier genome models since suspicious genomes with long branches can be spotted. In this work, four genomes showed extreme branch lengths, albeit they passed the genome quality thresholds. These four genomes come from different continents but have in common the instrument used for NGS data generation, the ION TORRENT. This instrument has shown to have higher mutation rates than Illumina instruments, supporting the hypothesis that these longer branches are more likely associated with sequencing artifacts rather than real higher evolution rates (Quail et al., 2012). This observation implies that genome assembly statistics alone are not enough for quality control. Complementary phylogenomic analyses help to detect genome assemblies that could accumulate higher error rates.

In general, the analyzed *C. hominis* genomes have low alternate allele counts, 85% with less than 470 events, suggesting that most of the infected individuals might harbor one clonal *Cryptosporidium* genome with minor nucleotide variants (0.005%).

The highest number of alternate alleles went as high as 112,225, nearly 34 times higher than the observed median value. In this specific patient, we might be dealing with a case of *Cryptosporidium* mixed species infection. This observation is supported by the facts that the global genome identity went down to 98.96% and that the *Cryptosporidium* assembled genome size was exceptionally large, exceeding 11 Mbp.

Several research works have described the presence of mixed *C. hominis* genotypes or even species in one infected individual (Cama et al., 2006; Gilchrist et al., 2018; Korpe et al., 2019; Sannella et al., 2019). These observations can be reconciled with our results considering that coinfecting *C. hominis* genotypes of other *Cryptosporidium* species could be below the detection threshold of the actual metagenomic approaches.

The phylogenomic analysis allowed us to discover two *C. hominis* lineages with continental segregation. These lineages were named based on the observed continental distribution bias as *C. hominis* Euro-American (EA) lineage and the *C. hominis* Afro-Asian (AA) lineage.

The reference isolate UDEA01 is part of the EA lineage, as well as the other Latin-American isolates UdeAa567 and SWEH12. This clade also encompasses isolates of the United States, the United Kingdom, Spain, and Greece. Only one genome belongs to a different continental region, Afr10, which was isolated from a human in Madagascar, Africa. All the genomes in the EA clade are classified as genotypes IbA10G2/IbA12G2 except for *C. hominis* UDEA01, which has been classified as genotype IeA11G3T3. Using an SNVs phylogenomic approach, Sikora et al. (2017) made a previous observation that supports our findings. In their study, *C. hominis* genomes of the genotype IbA10G2 separated in a monophyletic clade independent of other studied *C. hominis* genotypes. Another line of evidence that supports our finding was published recently by Tichkule et al. In their work, the researchers observed a geographic structuring of the *C. hominis* isolates in African countries (Tichkule et al., 2021).

Haplotype network analysis supported the geographical isolation of the EA and AA *C. hominis* lineages. Additionally, the AA lineage showed a higher haplotype diversity. Nevertheless, it must be highlighted that the sampling was higher in the AA lineage.

INDEL events are frequent in *Cryptosporidium* genomes (Arias-Agudelo et al., 2020). PCA analysis of the INDEL profiles showed that they can segregate *C. hominis* of the other studied *Cryptosporidium* species: *C. parvum*, *C. meleagridis*, and *C cuniculus*. Additionally, INDEL events profile showed to be taxonomically informative in *C. hominis* since they were able to segregate the two lineages, adding support to the previous observe results.

Summarizing, three independent lines of evidence support our observation that *C. hominis* encompasses two separate lineages. These two monophyletic lineages have a differential continental distribution pattern with a clear dominance of one lineage in Eastern Europe and the Americas, while the other is the main lineage in Africa and Asia.

Previous research works on infectious diseases have demonstrated a phylogeographic relationship among European and American human populations. This is the case for the etiological agent of human tuberculosis, *Mycobacterium tuberculosis*. This pathogen presents a specific lineage, called the LAM family (L4.3), which is more common in the Mediterranean and Latin-American countries, and showed the spread of the bacteria associated with human migrations from Europe to America (Gagneux et al., 2006; Brynildsrud et al., 2018).

By the beginning of the 2000s *C. parvum* and *C. hominis* were considered a single species (Sulaiman et al., 2000), with the name of the former as the type species. Two years later, Morgan-Ryan et al., using complementary molecular analysis showed that, in fact, two closely related species could be discriminated and *C. hominis* and *C. parvum* were separated as independent species (Morgan-Ryan et al., 2002).

Ending the next decade, in 2019, within the *C. parvum* clade, a new subspecies was discovered, *C. parvum antroponosum* (Nader et al., 2019). *Cryptosporidium parvum* parvum is described as a zoonotic parasite while the new subspecies *C. parvum* antroponosum has an anthroponotic transmission scheme. These discoveries were achieved thanks to the incorporation of more resolutive molecular analytical methods, like phylogenomic tools.

In this work, we present a similar observation for *C. hominis*, this new lineage may represent a new subspecies, but its confirmation and biological implications should be further investigated. Previous evolutionary works have shown an evolution model in *Cryptosporidium* parasites where speciation events are related to new host adaptations. In almost every major vertebrate lineage, an adapted *Cryptosporidium* species have been described (Garcia-R and Hayman, 2016). In the case of *C. hominis*, a particularly interesting question raises since *C. hominis* has been already adapted to our human ancestors around 6 million years ago (Garcia-R and Hayman, 2016). Additional work should be performed in order to establish the origin of the separation of the *C. hominis* lineages.

## Data Availability

The datasets presented in this study can be found in online repositories (NCBI SRA). See Supplementary Table S1. New Colombian C hominis genome read data were deposited in the NCBI SRA database accession number SRR14522748 (https://dataview.ncbi.nlm.nih.gov/object/SRR14522748).
