# Supplementary material for: Cryptosporidium hominis Phylogenomic Analysis Reveals Separate Lineages With Continental Segregation
Source: Front Genet. 2021 Oct 14;12:740940. doi: 10.3389/fgene.2021.740940 (PMC8552020; doi:10.3389/fgene.2021.740940)
Supplement: Supplementary file 2 [file DataSheet1.docx]

**SUPPLEMENTARY MATERIAL**

***Cryptosporidium hominis* phylogenomic analysis reveals separate lineages with continental segregation**

Felipe Cabarcas^1,2^, Ana Luz Galvan-Diaz^3^, Laura M. Arias^1^, Gisela María García-Montoya^4^, Juan M. Daza^5^ & Juan F. Alzate^1,4,*^.

^1^ ﻿Centro Nacional de Secuenciación Genómica - CNSG, Sede de Investigación Universitaria – SIU. Departamento de Microbiología y Parasitología, Facultad de Medicina, Universidad de Antioquia. Medellín, Antioquia, Colombia

^2^ Grupo SISTEMIC, Departamento de Ingeniería Electrónica, Facultad de Ingeniería, Universidad de Antioquia. Medellín, Antioquia, Colombia

^3^ Environmental Microbiology Group, School of Microbiology, Universidad de Antioquia, Medellin, Antioquia, Colombia

^4^ ﻿Grupo Pediaciencias. Facultad de Medicina, Universidad de Antioquia. Medellín, Antioquia, Colombia

5 Grupo Herpetológico de Antioquia, Institute of Biology, Universidad de Antioquia, Calle 70 No. 52-21, Postal Code 050010, Medellín, Colombia

*Corresponding Author:

Juan F. Alzate. Carrera 53 # 61 – 30, Medellín, Antioquia, 050010, Colombia

Email address: jfernando.alzate@udea.edu.co

**Supplementary methods**

**Python scripts**

[In-house COUNT_FASTA: countFasta.py ]

#!/usr/bin/env python

# countFasta

# AUTHOR: Felipe Cabarcas

# LAST REVISED: June 2021

#

# Centro Nacional de Secuenciacion Genomica --CNSG, Universidad de Antioquia

# All rights reserved.

# This program gets basic statistics from a fasta file

import os,argparse

from Bio import SeqIO

if __name__ == '__main__':

parser = argparse.ArgumentParser(description='Get Fasta file stats')

parser.add_argument('-f', '--f', help='Fasta File', required=True)

args = parser.parse_args()

Fasta = SeqIO.parse(args.f, "fasta")

FastaSize = []

for Seq in Fasta:

FastaSize.append(len(Seq.seq))

HalfSize = sum(FastaSize)/2

sumSize = 0

N50 = 0

for val in sorted(FastaSize):

sumSize += val

N50 = val

if sumSize > HalfSize:

break

print('Total length of sequence:\t',sum(FastaSize))

print('Total number of sequences:\t',len(FastaSize))

print('Average contig length is:\t',int(sum(FastaSize)/len(FastaSize)))

print('Largest contig:\t\t\t',max(FastaSize))

print('Shortest contig:\t\t',min(FastaSize))

print('N50 stats:\t\t\t',N50)

[In-house NUC-DIFF indels results: nucDiffgff2bed.py]

#!/usr/bin/env python

# AUTHOR: Felipe Cabarcas

# LAST REVISED: June 2021

#

# Centro Nacional de Secuenciacion Genomica --CNSG, Universidad de Antioquia

# All rights reserved.

# This program gets summary of insertions and deletions from a list

# of nucDiff results files

import os,argparse,ntpath

import statistics

def createFile(Table, Lista, fileName):

count = 0

completeTable = []

ofile = open(fileName,'w')

for key in Table:

inList = [key, len(Table[key])]

for genome in Lista:

if genome in Table[key]:

inList.append(1)

else:

inList.append(0)

completeTable.append(inList)

line = "Gene_St_Ed_Sz\tNONzeros\t"+'\t'.join(str(x) for x in Lista)

ofile.write(line+'\n')

for key in completeTable:

line ='\t'.join(str(x) for x in key)

ofile.write(line+'\n')

ofile.close()

def readAnnotation(fileName):

fileIt = open(fileName)

genes = {}

for line in fileIt:

if not line.startswith('#'):

temp = line.split('\t')

if temp[2] == 'protein_coding_gene':

sequence = temp[0]

start = int(temp[3])

end = int(temp[4])

name = temp[8].split(';')[0].split('=')[1]

if sequence in genes:

genes[sequence].append([start,end,name])

else:

genes[sequence] = [[start,end,name]]

fileIt.close()

return genes

def isInGene(genTable,seqId,coord):

pos = int(coord.split('-')[0])

lista = genTable[seqId]

isIn = False

for item in lista:

if pos >= item[0] and pos<=item[1]:

isIn = True

break

return isIn

if __name__ == '__main__':

parser = argparse.ArgumentParser(description='Create Summary Table of genome stats from nucdiff')

parser.add_argument('-c', '--c', help='NucDiff results base Dir', required=True)

parser.add_argument('-r', '--r', help='ref annotation', required=False)

args = parser.parse_args()

annotationTable = None

if args.r:

annotationTable = readAnnotation(args.r)

deletion = 'deletion'

insertion = 'insertion'

baseDir = args.c

tableDel = {}

tableIns = {}

Lista = []

for file in os.listdir(baseDir):

Gname = file.split('vs')[1]

Lista.append(Gname)

gfffile = open(baseDir+'/'+file+'/results/'+file+'_query_snps.gff')

for line in gfffile:

if not line.startswith('#'):

temp = line.strip().split('\t')[8].split(';')

featuretype = temp[1].split('=')[1]

size = temp[2].split('=')[1]

sequence = temp[4].split('=')[1]

coordenates = temp[5].split('=')[1]

inGenes = True

if annotationTable:

inGenes = isInGene(annotationTable,sequence,coordenates)

if inGenes:

if featuretype == deletion and int(size)>=1:

start = coordenates.split('-')[0]

end = coordenates.split('-')[1]

field = '_'.join([sequence,start,end,size])

if field in tableDel:

tableDel[field].append(Gname)

else:

tableDel[field] = [Gname]

elif featuretype == insertion and int(size)>=1:

start = coordenates

end = coordenates

field = '_'.join([sequence,start,end,size])

if field in tableIns:

tableIns[field].append(Gname)

else:

tableIns[field] = [Gname]

gfffile.close()

Lista.sort()

createFile(tableDel, Lista, "nucMAT_deletions.tab")

createFile(tableIns, Lista, "nucMAT_insertions.tab")

[In-house PCA indels graph: completeSummary.py]

#!/usr/bin/env python

# AUTHOR: Felipe Cabarcas

# LAST REVISED: June 2021

#

# Centro Nacional de Secuenciacion Genomica --CNSG, Universidad de Antioquia

# All rights reserved.

# This program creates PCA of insertions and deletions based on nucDiff filtered results

import pandas as pd

import numpy as np

import argparse

import matplotlib

import matplotlib.pyplot as plt

import scipy

import scipy.cluster.hierarchy as sch

from sklearn import cluster

from sklearn.preprocessing import StandardScaler

from sklearn.decomposition import PCA

from matplotlib.lines import Line2D

def getGroups(df,species,Labels):

groups = []

for spe in species:

if spe[0:4] == 'Chom':

continent = spe.split('_')[1]

groups.append(continent)

else:

groups.append(spe[0:4])

return groups

def pca(df, groups, groups2, labeltargets,labelmarker, Out):

features = list(df.columns)

markers = {'C. hominis AA':'^','C. hominis EA':'s','C. cuniculus':'o', 'C. meleagridis':'o','C. parvum':'o'}

allColors={'AFRICA':'goldenrod', 'AMERICA':'yellowgreen','ASIA':'tomato','EU':'cornflowerblue','OCEANIA':'darkgrey','Ccun':'blue', 'Cmel':'fuchsia','Cpar':'darkgreen'}

legend_elementsC = [Line2D([0], [0], marker='o', color='w', label=labeltargets[0] ,markerfacecolor='goldenrod', markersize=10),

Line2D([0], [0], marker='o', color='w', label=labeltargets[1],markerfacecolor='yellowgreen', markersize=10),

Line2D([0], [0], marker='o', color='w', label=labeltargets[2],markerfacecolor='tomato', markersize=10),

Line2D([0], [0], marker='o', color='w', label=labeltargets[3],markerfacecolor='cornflowerblue', markersize=10),

Line2D([0], [0], marker='o', color='w', label=labeltargets[4],markerfacecolor='darkgrey', markersize=10),

Line2D([0], [0], marker='o', color='w', label=labeltargets[5],markerfacecolor='blue', markersize=10),

Line2D([0], [0], marker='o', color='w', label=labeltargets[6],markerfacecolor='fuchsia', markersize=10),

Line2D([0], [0], marker='o', color='w', label=labeltargets[7],markerfacecolor='darkgreen', markersize=10)]

legend_elementsL = [Line2D([0], [0], marker='^', color='w', label='C. hominis AA',markerfacecolor='k', markersize=10),

Line2D([0], [0], marker='s', color='w', label='C. hominis EA',markerfacecolor='k', markersize=10)]

x = df.loc[:, features].values

# Standardizing the features

x = StandardScaler().fit_transform(x)

pca = PCA(n_components=2)

principalComponents = pca.fit_transform(x)

precision = pca.get_precision()

print(precision)

print("PCA Explained Variance Ratio:", pca.explained_variance_ratio_)

principalDf = pd.DataFrame(data = principalComponents

, columns = ['principal component 1', 'principal component 2'])

finalDf = principalDf

finalDf['target'] = groups

finalDf['marker'] = groups2

fig = plt.figure(figsize = (8,8))

ax = fig.add_subplot(1,1,1)

ax.set_xlabel('Principal Component 1 ('+str(round(pca.explained_variance_ratio_[0]*100,2))+'%)', fontsize = 15)

ax.set_ylabel('Principal Component 2 ('+str(round(pca.explained_variance_ratio_[1]*100,2))+'%)', fontsize = 15)

for index, row in finalDf.iterrows():

ax.scatter(row['principal component 1']

, row['principal component 2']

, c = allColors[row['target']]

, marker = markers[row['marker']]

, s = 50)

legend1 = ax.legend(handles=legend_elementsC,loc="upper right")

ax.add_artist(legend1)

ax.legend(handles=legend_elementsL,loc="lower right")

ax.grid()

plt.savefig(Out+'_pca.pdf', dpi=600)

def plotMat(bio_df,outFile):

species = list(bio_df.index)

print("Num Species "+str(len(species)))

bio_array = bio_df.to_numpy(dtype=float)

current_cmap = matplotlib.cm.get_cmap('Greys') #RdBu

CS = plt.imshow(bio_array, cmap=current_cmap)#, vmin=-1, vmax=1)

plt.savefig(outFile+'.png', dpi=1000)

def leerTabla(fileName,outFile):

all_df = pd.read_csv(fileName, sep='\t',header=0,index_col=0)

all_df = all_df[all_df['NONzeros'] >= 5]

all_df = all_df.sort_values(by=['NONzeros'])

all_df = all_df.drop('NONzeros',axis=1)

transposed = all_df.T

return transposed

def common(df,al,el,Out):

logic_df = df.T

baseName = "allChom"

filt = al[1:]+el[1:]

filt_df = logic_df[filt]

filt_df['suma'] = filt_df.sum(axis=1)

logic_df = filt_df[filt_df['suma'] != len(filt)]

logic_df = logic_df.drop('suma',axis=1)

filt_df = filt_df[filt_df['suma'] == len(filt)]

filt_df = filt_df.drop('suma',axis=1)

filt_df = filt_df.drop(filt,axis=1)

filt_df.to_csv(Out+'_'+baseName+'_Common.csv')

baseName = al[0].split()[-1]

filt = al[1:]

filt_df = logic_df[filt]

filt_df['suma'] = filt_df.sum(axis=1)

filt_df = filt_df[filt_df['suma'] == len(filt)]

filt_df = filt_df.drop('suma',axis=1)

filt_df = filt_df.drop(filt,axis=1)

filt_df.to_csv(Out+'_'+baseName+'_Common.csv')

baseName = el[0].split()[-1]

filt = el[1:]

filt_df = logic_df[filt]

filt_df['suma'] = filt_df.sum(axis=1)

filt_df = filt_df[filt_df['suma'] == len(filt)]

filt_df = filt_df.drop('suma',axis=1)

filt_df = filt_df.drop(filt,axis=1)

filt_df.to_csv(Out+'_'+baseName+'_Common.csv')

def getGroups2(df,species,al,el,par,mel,cun):

groups = []

for spe in species:

if spe[0:4] == 'Chom':

if spe in al:

groups.append(al[0])

elif spe in el:

groups.append(el[0])

else:

print("Warning: "+spe+", not in the target species")

groups.append(spe[0:4])

elif spe[0:4] == 'Ccun':

groups.append(cun)

elif spe[0:4] == 'Cmel':

groups.append(mel)

elif spe[0:4] == 'Cpar':

groups.append(par)

else:

print("Error!!!!")

return groups

if __name__ == '__main__':

parser = argparse.ArgumentParser(description='Creates PCA of nucdiff indels')

parser.add_argument('-f', '--f', help='csv Files', required=True)

parser.add_argument('-la', '--la', help='linaje Americano', required=False)

parser.add_argument('-le', '--le', help='linaje Europeo', required=False)

parser.add_argument('-o', '--o', help='Out base files', default='temp')

args = parser.parse_args()

outBaseName='AnalisisTodos'

df = leerTabla(args.f,outBaseName)

species = list(df.index)

Afro = ["C. hominis AA"]

if args.la:

with open(args.la) as fp:

for line in fp:

Afro.append(line.strip())

Eur = ["C. hominis EA"]

if args.le:

with open(args.le) as fp:

for line in fp:

Eur.append(line.strip())

groups2 = getGroups2(df,species,Afro,Eur,'C. cuniculus','C. meleagridis','C. parvum')

labels = ['C. hominis (Africa)', 'C. hominis (America)','C. hominis (Asia)','C. hominis (Europe)','C. hominis (Oceania)','C. cuniculus','C. meleagridis','C. parvum']

labels2 = ['C. hominis AA', 'C. hominis EA','C. cuniculus', 'C. meleagridis','C. parvum']

groups = getGroups(df,species,labels)

pca(df,groups,groups2,labels,labels2,args.o)

common(df,Afro,Eur,args.o)

[CDSs in genomes: CDSinGenome.py]

#!/usr/bin/env python

# AUTHOR: Felipe Cabarcas

# LAST REVISED: June 2021

#

# Centro Nacional de Secuenciacion Genomica --CNSG, Universidad de Antioquia

# All rights reserved.

# This program gets the CDSs for a number of genomes, using a reference

#

import os,argparse

from Bio import SeqIO

from Bio import SearchIO

from Bio.SeqRecord import SeqRecord

from subprocess import Popen, PIPE

import shlex

def getBestAlign(aligFileName):

blast_result = SearchIO.read(aligFileName, 'blast-xml')

BestAlign = None

for res in blast_result:

maxBitScore = 0

BestAlign = None

for hsp in res.hsps:

if maxBitScore < hsp.bitscore:

maxBitScore = hsp.bitscore

BestAlign = hsp

return BestAlign

def launchBlastn(CDs,genome,outXMLFile,blastp):

if blastp:

line = "blastp -query "+CDs+" -subject "+genome+" -num_alignments 1 -outfmt 5 -out "+outXMLFile

else:

line = "blastn -query "+CDs+" -subject "+genome+" -num_alignments 1 -outfmt 5 -out "+outXMLFile

print(line)

Line = shlex.split(line)

process = Popen(Line, stdout=PIPE, stderr=PIPE)

(output, err) = process.communicate()

exit_code = process.wait()

if err:

if 'Warning' in str(err):

return True

else:

print(err)

return False

else:

return True

if __name__ == '__main__':

parser = argparse.ArgumentParser(description='Find CDs in Genome')

parser.add_argument('-c', '--c', help='Fasta File with cds', required=True)

parser.add_argument('-g', '--g', help='Fasta File with genome', required=True)

parser.add_argument('-o', '--o', help='Out Base File name', required=True)

parser.add_argument('-d', '--d', help='Out Dir Fasta File', required=True)

parser.add_argument('-p', '--p', help='Blastp', action='store_true', default=False)

parser.add_argument('-extra', '--extra', help='Extra bases before and after to be considered', default=0,type=int )

args = parser.parse_args()

CDss = SeqIO.parse(args.c, "fasta")

genome_fasta = SeqIO.to_dict(SeqIO.parse(args.g, "fasta"))

genomeMultifastaCDs = args.d+'/CDSs_'+args.o+'.fasta'

tempXML = "tempXml.xml"

tempCDs = "tempCDs.fasta"

FastaCDsGenome = []

Stats = {'CDS':0,'Found':0,'short':0,'sameSize':0,'larger':0}

print(args.o+'\n')

for CDS in CDss:

Stats['CDS'] += 1

with open(tempCDs,"w") as fw:

SeqIO.write (CDS,fw,"fasta")

succesfull = launchBlastn(tempCDs,args.g,tempXML,args.p)

alignBest = None

if succesfull:

alignBest = getBestAlign(tempXML)

else:

print("Could not align ",CDS.id)

if alignBest:

Stats['Found'] += 1

start = min(alignBest.hit_range[0],alignBest.hit_range[1])-args.extra

end = max(alignBest.hit_range[0],alignBest.hit_range[1])+args.extra

if args.p:

newseq = genome_fasta[alignBest.hit_id].seq[:]

else:

newseq = genome_fasta[alignBest.hit_id].seq[start:end]

newid = CDS.id

record = SeqRecord(newseq,id=newid,description=args.o)

FastaCDsGenome.append(record)

if len(record.seq) < len(CDS.seq):

Stats['short'] += 1

elif len(record.seq) == len(CDS.seq):

Stats['sameSize'] += 1

else:

Stats['larger'] += 1

else:

print("Warnning: ",CDS.id," Not Found in Genome")

with open(genomeMultifastaCDs,"w") as fw:

for record in FastaCDsGenome:

SeqIO.write ( record,fw,"fasta")

for key in Stats:

print(key,' : ',Stats[key])

[Creates a fasta file for each CDS from all genomes: createFasta4tree.py]

#!/usr/bin/env python

# AUTHOR: Felipe Cabarcas

# LAST REVISED: June 2021

#

# Centro Nacional de Secuenciacion Genomica --CNSG, Universidad de Antioquia

# All rights reserved.

# This program Creates a fasta file for each CDS from all genomes

#

import os,argparse

from Bio import SeqIO

from Bio.Seq import Seq

from Bio import SearchIO

from Bio.SeqRecord import SeqRecord

from subprocess import Popen, PIPE

import shlex

if __name__ == '__main__':

parser = argparse.ArgumentParser(description='Group CDs in single fasta')

parser.add_argument('-c', '--c', help='Fasta File with cds per genome', nargs='+', required=True)

parser.add_argument('-r', '--r', help='Ref File with all cds', required=True)

args = parser.parse_args()

dirCDS={}

genome_fasta = SeqIO.parse(args.r, "fasta")

for seqCds in genome_fasta:

dirCDS[seqCds.id] = []

for genomeCDs in args.c:

genome_fasta = SeqIO.to_dict(SeqIO.parse(genomeCDs, "fasta"))

for seqCds in dirCDS:

if seqCds in genome_fasta:

dirCDS[seqCds].append(genome_fasta[seqCds])

else:

temp = genomeCDs.split('CDSs_')

temp1 = temp[1].split('.')

dirCDS[seqCds].append(SeqRecord(Seq(''),id=seqCds,description=temp1[0]))

for fastaCDs in dirCDS:

with open("PerCDS/"+fastaCDs+".fasta","w") as fw:

for record in dirCDS[fastaCDs]:

SeqIO.write ( record,fw,"fasta")

**STEP by STEP bioinformatic Protocol.**

# Step by step analysis.

# For each C. hominis experiment the following steps were taken. Lets assume the first reads name was ERR970586.

# We star the the files ERR970586_1.fastq.gz and ERR970586_2.fastq.gz in READS_DIR

# Note that Iontorrent reads only have *_1.gz furthermore the reads from experiment SRR1558150, the second read was not used so it is assembled and analized as a single read genome

# Cleanning, this command produces ERR970586_1.fastq and ERR970586_2.fastq on current directory

rapifilt -l 30 -r 30 -w 2 -m 50 -i READS_DIR/ERR970586_1.fastq.gz READS_DIR/ERR970586_2.fastq.gz -o ERR970586

#In case of Iontorrent the line is

#rapifilt -l 30 -r 30 -w 2 -m 50 -fastq READS_DIR/READ_1.fastq.gz -o READ

# Assembly

spades.py --careful -t 40 -m 200 -k 33,55,77,99 -1 ERR970586_1.fastq -2 ERR970586_2.fastq -o SPADES_ERR970586

#In case of Iontorrent the line is

#spades.py --careful -t 40 -m 200 -k 33,55,77,99 -s READ.fastq -o SPADES_READ

# selectContigs from assembly

# Prior to this step it is necesary to index CptDB51CparvumIowaII.fasta for blast. It is the C. parvum Iowa II assembly

blastn -query SPADES_ERR970586/scaffolds.fasta -db CptDB51CparvumIowaII.fasta -outfmt 6 -evalue 1e-30 -num_alignments 5 > temp_blast.out

# Make a list of contigs with hit to Iowa II with a score greater that 300 and longer that 1000 bases

awk '$12>300' temp_blast.out | awk -F'_' '$4>1000' | awk '{print $1}' | sort -k1,1 | uniq > list.tab

# Select contigs in the list, using an in-house fasta selector C program

selectFastX -fasta SPADES_ERR970586/scaffolds.fasta -fastX_sel -list list.tab > ERR970586.fasta

# Stats: TotalBases LargestScaffold N50 NumScaffolds, were obtained with in-house python script [In-house COUNT_FASTA]

countFasta.py -f ERR970586.fasta

#After Assembling all, ERR970586.fasta will be copied to Chom_AMERICA_UdeA01.fasta

#Compare genome with C. hominis UdeA01 genome

dnadiff -p ERR970586vsChom_AMERICA_UdeA01 Chom_AMERICA_UdeA01.fasta ERR970586.fasta

# Obtain AlignedBasesUdeA01 AvgIdentityUdeA01 TotalSNPsUdeA01 from ERR970586vsChom_AMERICA_UdeA01.report by

grep AlignedBases ERR970586vsChom_AMERICA_UdeA01.report |head -n 1 |awk '{print $3}'|awk -F'(' '{print $2}' |sed 's/)//g'

grep AvgIdentity ERR970586vsChom_AMERICA_UdeA01.report |head -n 1 | awk '{print $3}'

grep TotalSNPs ERR970586vsChom_AMERICA_UdeA01.report |head -n 1 | awk '{print $3}'

#mapping reads to the self assembly

bowtie2-build ERR970586.fasta scaffolds

bowtie2 -p 8 -x scaffolds -1 ERR970586_1.fastq -2 ERR970586_2.fastq | samtools view -F 3584 -b --threads 8 > ERR970586.bam

# In case of Iontorrent the line is

# bowtie2 -p 8 -x scaffolds -U READ.fastq | samtools view -F 3584 -b --threads 8 > READ.bam

samtools sort -o ERR970586_s.bam

samtools index ERR970586_s.bam

samtools faidx ERR970586.fasta

bcftools mpileup --redo-BAQ --min-BQ 30 --per-sample-mF --skip-indels --threads 8 --annotate FORMAT/AD,FORMAT/ADF,FORMAT/ADR,FORMAT/DP,FORMAT/SP,INFO/AD,INFO/ADF,INFO/ADR -f ERR970586.fasta ERR970586_s.bam | bcftools call --multiallelic-caller --variants-only -Ov | bcftools view -i '%QUAL>=30' > ERR970586.vcf

#To get the number of ALtSNPs the following python script was used

# mappingSNPs = open('ERR970586.vcf')

# ALtSNPs = 0

# for line in mappingSNPs:

# if not line.startswith('#'):

# ALtSNPs += 1

# print(ALtSNPs)

# Those genomes TotalBases >= 8167000 and ALtSNPs < 3825 Were considerered for the following analisis

# The coverage per contig it is obtained with

samtools coverage -o ERR970586_coverage.tab ERR970586_s.bam

# To get the median coverage the following python script was used

# coverageF = open('ERR970586_coverage.tab')

# depth = []

# for line in coverageF:

# if not line.startswith('#'):

# dato = float(line.strip().split('\t')[6])

# depth.append(dato)

# print(statistics.median(depth))

# We run busco for each genome

busco -m geno -l coccidia_odb10 -i ERR970586.fasta -o BUSCO_ERR970586

#We got the stats with

grep 'C:' BUSCO_ERR970586/short_*.txt |awk '{print $2}'| sed 's/C//g'|sed 's/://g'|sed 's/S//g'|sed 's/F//g'|sed 's/M//g'|sed 's/n//g'|sed 's/\[//g'|sed 's/\]//g'|sed 's/D//g'|sed 's/://g'|sed 's/,//g'|awk -F'%' '{printf("%f\t%f\t%f\t%f\t%f\n",$1,$2,$3,$4,$5)}'

# In order to create the phylogenetic tree, the CDS are selected using an in house script [CDSs in genomes: CDSinGenome.py] for all selected genomes, using GenesEvNeutralCparvum61.fasta which are the C. Parvum IowaII genes with Neutral evolution. It generates a fasta file with the CDSs of each Genome

CDSinGenome.py -c GenesEvNeutralCparvum61.fasta -g ERR970586.fasta -d CDS_DIR -o CDS_ERR970586.fasta

# To be able to align each CDS from all genomes together it is necesary to create a file per CDS with the name of the CDS (CDS_NAME.fasta)

./createFasta4tree.py -r GenesEvNeutralCparvum61.fasta -c [List of all CDS (fasta) creates in the previews step]

# for each CDS_NAME.fasta the CDSs are aligned with mafft

mafft --thread 2 --threadtb 2 --threadit 0 --inputorder --adjustdirection --anysymbol --auto CDS_NAME.fasta 1> aligned_CDS_NAME.fasta

# A file with all the aligned CDSs is created: aligned_CDS.txt, to be able to concatenate them usign catsequences, this program creates allseqs.fas and allseqs.partitions.txt

catsequences aligned_CDS.txt

# It is necesary to modify a partitions file to prepare it for iqtree

sed -e 's/.fasta//' -e 's/;//' -e 's/alignedCDS\///' allseqs.partitions.txt | awk '{print"DNA, "$1" "$2" "$3}' > allseqs.partitions

# The phylogenetic tree is created with the following line

iqtree2 -s allseqs.fas -spp allseqs.partitions -B 5000 -T AUTO -m MFP+MERGE -rcluster 10 -pre ALLGenomes

#Te get insertions and deletions, respect C. parvum IowaII

nucdiff Cpar_IowaII.fasta ERR970586.fasta Cpar_IowaIIvsERR970586 Cpar_IowaIIvsERR970586

# To get the summary of the results we used [In-house NUC-DIFF indels results] in the following NUCDIFF_GENOME it is a DIR with all the nocdiff results, and it generates nucMAT_deletions.tab and nucMAT_insertions.tab, while the AfroAsiaticLineage and EuroAmericanLineage were obtained from the tree manually. The following script generates nucMAT_deletions.tab and nucMAT_insertions.tab

./nucDiffgff2bed.py -c NUCDIFF_GENOME

# In order to get the PCA analisis,

./completeSummary.py -o delNucComplete -f nucMAT_deletions.tab -la AfroAsiaticLineage -le EuroAmericanLineage

./completeSummary.py -o insNucComplete -f nucMAT_insertions.tab -la AfroAsiaticLineage -le EuroAmericanLineage

**Complete Cryptosporidium Tree**
